# A Laser-Based Vision System for Weld Quality Inspection

**DOI:** 10.3390/s110100506

**Published:** 2011-01-06

**Authors:** Wei Huang, Radovan Kovacevic

**Affiliations:** Research Center for Advanced Manufacturing, Southern Methodist University, 3101 Dyer Street, Dallas, TX 75205, USA; E-Mail: huangw@smu.edu

**Keywords:** laser triangulation, non-destructive, inspection, weld quality

## Abstract

Welding is a very complex process in which the final weld quality can be affected by many process parameters. In order to inspect the weld quality and detect the presence of various weld defects, different methods and systems are studied and developed. In this paper, a laser-based vision system is developed for non-destructive weld quality inspection. The vision sensor is designed based on the principle of laser triangulation. By processing the images acquired from the vision sensor, the geometrical features of the weld can be obtained. Through the visual analysis of the acquired 3D profiles of the weld, the presences as well as the positions and sizes of the weld defects can be accurately identified and therefore, the non-destructive weld quality inspection can be achieved.

## 1. Introduction

Welding, as the most commonly used process for joining metals and plastic, is vital to the economy of a country because up to 50% of the gross national product is related to welding in one way or another [1]. However, welding is such a complex process that the visually recognizable quality of the weld is affected by a number of process variables. The potential weld defects greatly deteriorate the mechanical properties of the welded structures weld quality and as a result, the risks of part fatigue, failure even disaster are significantly increased. Therefore, in order to detect the possible presence of different weld defects, proper sensing, monitoring and inspection methods are necessary for quality control. Generally, there are two categories of methods that are applied for weld quality inspection: on-line methods and off-line methods. As welding is such a complex process, there are many different physical phenomena such as electromagnetic radiation, optical and acoustic emission and plasma generation that will occur. Along with these phenomena, different types of signals are generated during the welding process and usually these signals are closely associated with the weld quality. Therefore, different on-line sensing methods have been developed to monitor the weld quality and detect the weld defects based on the signatures of the signals acquired during the welding process. Fortunko *et al.* [2] applied an electromagnetic transducer to evaluate the quality of butt welds in a non-destructive way. Park *et al.* [3] used optical sensors such as infrared (IR) photodiodes and ultraviolet (UV) photodiodes to detect the spatters generated during CO_2_ laser welding. Gu *et al.* [4,5] developed a statistical approach to predict the weld quality based on the spectrum analysis of acoustic signals. Huang *et al.* [6,7] applied microphone as the sensor to monitor the depth of weld penetration based on the acquired and processed airborne sound signals and established a neural network model to predict the depth of weld penetration based on extracted acoustic signatures. A sensor to monitor the structure-borne acoustic emission is also employed to detect interior weld defects such as cracks, porosity, and weld penetration based on acoustic emission signals at a frequency from 50 to 900 kHz [8]. These on-line sensing methods developed to inspect the weld quality and detect the weld defects heavily rely on the efficient processing and accurate interpretation of the signals from the welding process. For the purpose of weld quality inspection, these on-line methods are sometimes not reliable if the signals are contaminated by noises from hostile industrial environment and misinterpreted. Besides on-line methods for weld quality inspection, a number of off-line methods are also available. For different external and internal weld defects, as shown in Table 1, there are different types of off-line inspection methods. For example, inspection methods based on ultrasonic, radiography (e.g., x-ray), and electrical eddy current and magnetic particle inspection are effective for detection of interior weld defects such as crack and porosity [9]. On the other hand, as non-destructive testing method, vision-based inspection systems are always employed to detect external defects such as reinforcement, root concavity, undercut, sharp corner, incomplete filled groove, root dropout, misalignment of the welded metal sheets, and partial penetration. Jeng *et al.* [10] used a CCD camera to inspect the gap and alignment for the laser butt joint welding. Ho *et al.* [9] applied a vision system to automatically detect the surface cracks in welds. Shafeek *et al.* [11] developed an expert vision system to inspect different weld defects during the gas pipeline welding. Among different types of vision systems for inspection purpose, the vision systems based on principle of laser triangulation attract much attention both from academia and industry. Laser triangulation method was initially developed for distance measurement in a one-dimensional space. Based on the function of distance measurement, laser triangulation sensors are also developed for other applications such as a novel pulse measurement system developed by Wu *et al.* [12]. By extending laser triangulation sensor into two-dimensional space, it also can be applied as 3D imaging and reconstruction sensors in different areas such as automotive [13], culture heritage, medicine, criminal investigation [14] and integration with robot for the purpose of measurement [15] and manufacturing industry.

Based on reported data, the feasibility of using vision system to inspect weld quality has already been studied. However, most of the vision systems themselves are usually complex systems consisting of expensive and bulky hardware and software. The cost of the vision system, the practical implementation difficulty of the vision system into industrial environment, and the image processing efficiency always bring up some challenges that greatly limit their applications. As shown in Table 2, the costs and features of different systems are compared. It can be observed that most of the systems cost much more than 10,000 dollars while only one laser scanner from NextEngine is lower than 10,000 dollar. However, it can be noted that this relatively cheap system is not developed for real-time industrial application. For example, the USB cable used by this system only has a maximum length of 0.5 m and the low scan speed and small amount of image data that can be processed per second are not suitable for industrial application with requirements on the hardware and real-time processing.

In order to solve these issues, a low-cost laser-based vision system is developed in this study to achieve the weld quality inspection efficiently. In order to satisfy the demand for weld quality inspection in industrial environment, a Gigabit Ethernet (GigE) industrial camera is adopted to overcome the disadvantages of the commercially available cameras. The GigE camera can transfer images at a high speed up to 196 frames per second with a cable length up to 100 m. These features of the GigE industrial camera well accommodate the requirements for the high speed welding of large structures in a hostile industrial environment and for the post-weld quality inspection. Additionally, as a standard yet flexible off-the-shelf development platform, LabVIEW is used in this study to develop the image processing module. The industry-friendly LabVIEW platform is more flexible and easier for engineers to develop a complex system than the proprietary hardware and software platforms based on C++ or other programming languages. Based on the graphic programming language and comprehensive ready-to-use functions and different modules for data operation, image processing, motion control, and user interface design, LabVIEW-based program can be easily customized for the purpose of weld quality inspection and other applications. In addition, a highly efficient yet easy-to-implement image processing algorithm is proposed and realized in this study to process the stream of images acquired by the GigE camera. By analyzing the obtained 3D profile of the weld, the position information as well as the geometrical features of the weld and the presence of weld defects can be accurately identified.

## 2. Design of the Laser-Based Vision Sensor

**Figure 1.**
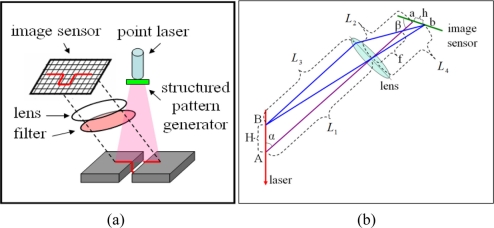
Design principle of the laser-based vision sensor: **(a)** schematic of the sensor and **(b)** design of sensor parameters based on laser triangulation.

As shown in Figure 1(a), a typical laser triangulation vision sensor consists of a laser generator, a structured pattern generator, an image sensor, a focus lens and an optical filter.

A high quality laser beam is generated by a low power diode laser. The laser beam passes through a structured pattern generator and a laser plane is generated and projected onto the surface of the weld, resulting in a laser stripe that follows the profile of the weld surface. The laser light is scattered by the weld surface and reflected back into different directions. As the image sensor, a complementary metal oxide semiconductor (CMOS) or a charge-coupled device (CCD) camera is used to collect the reflected laser light and therefore, the reflected laser strip that follows the profile of the weld is imaged back on the image sensor. In front of the image sensor, a focus lens is arranged at a specific distance and angle with respect to the image sensor to guarantee that the images of the laser stripe reflected from the weld at different distances are always focused on the image sensor. The distance between the vision sensor and the weld, as well as the lateral position and geometrical features of the weld can be precisely determined based on the mathematical relationship derived from geometrical optics as illustrated in Figure 1(b).

As shown in Figure 1(b), the design principle of the laser-triangulation-based vision sensor developed in this study will be first illustrated in the one-dimensional space. A laser beam is projected vertically onto the target surface at point “A”, and the reflected light from point “A” is captured by the image sensor and the image of point “A” is formed at point “a” on the image sensor. The point “A” is the intersection point of the laser beam and the optical axis of the focus lens. When the position of the target changes to point “B”, the image of point “B” is formed at point “b” on the image sensor. The distance between points “A” and “B” is *H* and the distance between points “a” and “b” is *h*. *L*_1_ is the object distance of point “A” with respect to the lens while *L*_2_ is the image distance of point “a”. *α* is the angle between the optical axis and the laser beam and *β* is the angle between the optical axis and the image sensor. In order to measure the distance change *H* between points “A” and “B” based on the images of points “a” and “b”, the relationship between *H* and *h* should be established based on geometrical optics. In order to obtain this relationship, the object distance *L*_3_ of point “B” and the image distance *L*_4_ of point “b” can be easily derived:

(1) $$L_{3}=L_{1}-H\;\text{cos}\;\alpha$$

(2) $$L_{4}=L_{2}+h\;\text{cos}\;\beta$$

According to the law of image focusing, the following relationship is obtained, where *f* is the focal length:

(3) $$\frac{1}{L_{1}}+\frac{1}{L_{2}}=\frac{1}{L_{3}}+\frac{1}{L_{4}}=\frac{1}{f}$$

Based on Equations (1–3), Equations (4) and (5) can be derived:

(4) $$L_{2}=\frac{L_{1}f}{L_{1}-f}$$

(5) $$L_{4}=\frac{L_{3}f}{L_{3}-f}=\frac{\left(L_{1}-H\;\text{cos}\;\alpha \right)\;f}{L_{1}-H\;\text{cos}\;\alpha -f}$$

According to the law of image magnification, Equation (6) can be obtained:

(6) $$\frac{H\;\text{sin}\;\alpha }{h\;\text{sin}\;\beta }=\frac{L_{4}}{L_{3}}$$

By combining Equations (1–6), the relationships between *H* and *h* and *α* and *β* can be established as:

(7) $$H=\frac{h\;\text{sin}\;\beta \left(L_{1}-f\right)}{f\;\text{sin}\;\alpha -h\;\text{cos}\;\alpha \text{sin}\beta }$$

(8) $$\beta ={\text{tan}}^{-1}\left(\frac{L_{1}-f}{f}\text{tan}\;\alpha \right)$$

Based on the equations derived above, the optical parameters such as *L*_1_, *L*_2_, *α*, *β* and *f* can be designed correspondingly. The change of the target distance H can be measured by the relationship described by Equation (7). By extending the relationship associated with the law of image magnification into the two-dimensional space, the width of the target could also be calculated based on the obtained images. Since the approach to mathematically model the vision sensor is purely a geometric approach assuming thin lens, the errors induced by the effect of refraction and distortion of the lens will be compensated by a sensor calibration process introduced in Section 4.

**Figure 2.**
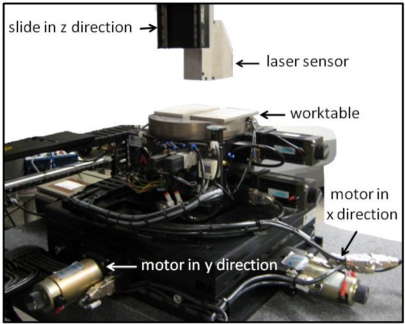
Fabricated laser-based vision sensor mounted on a multi-axis motion system.

As shown in Figure 2, the fabricated vision sensor incorporates a structured-light laser generator, a GigE camera, a lens and an optical filter. The structured-light laser generator perpendicularly projects a laser plane with a wavelength of 657.9 nm onto the target weld. To acquire the image of the formed laser stripe that follows the profile of the target weld, a GigE camera with an embedded CMOS image sensor is arranged at the designed angle of β with respect to the optical axis of the lens and a distance of *L*_2_ with respect to the lens. The method to assure the assembly accuracy of all the sensor components is first to sketch the pre-design geometrical arrangement of the components with taking consideration of their geometric specification with CAD software. Then different tooling machines are used to process the metal plates to form the sensor housing based on the CAD sketch. With accurate positions of screw holes on the metal housing, the sensor components are then fixed to the metal housing in predefined angles and positions. The CMOS image sensor is a monochromatic sensor chip with a 659 × 494 array of eight-bit pixels. It can be used to acquire grayscale image with a light intensity ranging from 0 to 255, while 0 represents the lowest light intensity, and 255 represents the highest light intensity. The frame rate of the GigE camera is up to 196 frames per second, and the cable length is up to 100 m. In front of the camera, the focus lens with a focal length f is also precisely arranged at the designed distance of *L*_1_ with respect to the intersection point between the laser beam and the optical axis. The angle between the optical axis and the laser stripe is arranged at the designed angle of *α*. In addition, a narrow-band optical filter centered at 658 nm is placed in front of the focusing lens. This optical filter will block out extraneous light generated during the welding process. Therefore, only the reflected laser stripe in the wavelength of 657.9 nm can pass through the optical filter to the image sensor while light disturbance and noise in other wavelengths will be filtered out. By precisely arranging different components inside the vision-based sensor module at the designed optical parameters, the position information and the geometrical features of the target weld can be obtained based on the mathematical relationship derived above. As shown in Figure 2, the fabricated laser-based vision sensor is mounted on a vertically movable slide above the worktable of the multiple-axis motion system. In order to perform weld quality inspection, the motion system is controlled to achieve movement in 3D space and the profile of the weld then can be scanned along specific direction. In this study, the images acquired by the vision sensor are transferred to a computer through an Ethernet cable. A developed image processing module that will process the images and extract useful quality information about the weld is detailed in the following section.

## 3. Design of the Image Processing Module

In order to perform the weld quality inspection and obtain the position information and geometrical features of the weld defects, the image of the laser stripe that follows the profile of the weld needs to be extracted first. According to previous researches, there are different methods for laser stripe extraction, such as Gaussian approximation, Center of mass, Linear approximation, Blais and Rioux detector, Parabolic estimator [16]. For the purpose of weld quality inspection, the inspected weld surface is usually a lambertian surface, for which scenario the laser stripe extraction is not as complex as other types of surfaces such as specular surface and translucid surface [17]. Therefore, the method to extract the laser stripe used in this study is easy to achieve yet effective and accurate for the purpose of weld quality inspection. The acquired digital grayscale image can be considered as a pixel array in a size of 659 × 494. In order to lower the computational cost, a region of interest (ROI) with m rows and n columns of pixels can be selected before further processing. After the ROI is chosen, the light intensity I(i, j) for each pixel in the ROI can be determined. For each pixel, there is a corresponding grayscale value of the light intensity ranging from 0 to 255. In order to remove the noise of high frequency in the image, the light intensity I(i, j) at each pixel is averaged with neighboring pixels by a Gaussian filter with a kernel size of 7 × 7. The Gaussian filter is used to perform noise reduction for the images. The kernel size is chosen based on the different performances of image noise reduction at different kernel sizes such as 3 × 3, 5 × 5 and 7 × 7. The size used has a better performance to remove noises in the image. By considering the acquired image consisting of m rows and n columns of pixels, the pixel with the maximum light intensity in each column of the pixel array is the one on the intersection between the vertical column of pixels and the horizontal row of pixels that represent the laser stripe. By searching for the pixel with the maximum light intensity at each column of the pixel array, a collection of such pixels that represent the laser stripe can be obtained. Since the laser stripe projected on the target has a thickness, more than one pixel in each column has the maximum light intensity. Therefore, the middle one of those pixels with the maximum light intensity is searched as the desired pixel to be extracted. After the image of the laser stripe is extracted, the position information of each pixel on the extracted laser stripe can be calculated based on the mathematical relationship derived in Section 2. The test result of this image processing algorithm indicates that this method only takes about 3 milliseconds to process each image and more than 300 images can be processed in a second, which is efficient yet easy to implement. And by comparing the laser stripe extraction results from this simple method detailed above and the most commonly used method Center of Mass, only very subtle difference between each other can be observed. Due to the simplicity and adequate accuracy, this method is applied to perform laser stripe extraction in this study.

**Figure 3.**
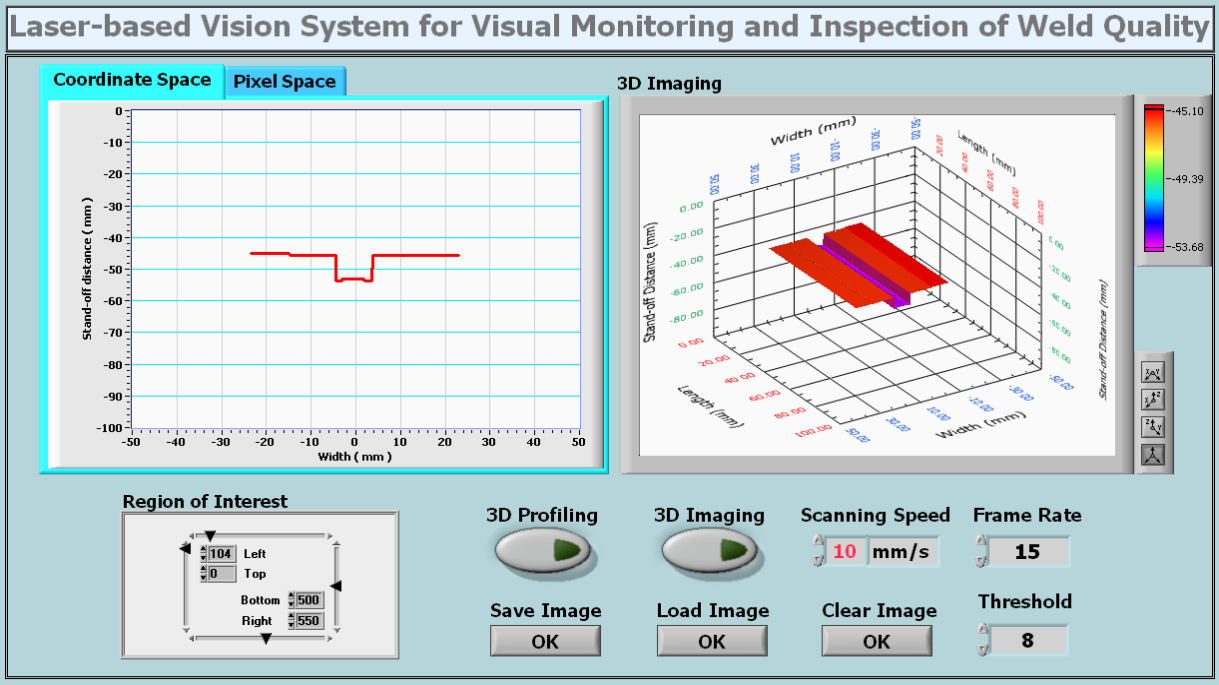
Human-machine interface of the laser-based vision system.

In order to visualize the image processing results and control some of the parameters related to the image processing module, a human-machine interface is designed as shown in Figure 3. At the top-left corner of the interface, it shows the weld bead profile both in the coordinate space and pixel space. In the coordinate space, the position information and geometrical features of the traversal weld profile with respect to the vision sensor such as the stand-off distance and width are available. At the top-right corner of the interface, the 3D profile of the weld along with its position information and geometrical features can be obtained when the vision sensor scans across the weld bead surface by using the motion system. At the bottom-left corner, the region of interest of the image can be chosen to minimize the computational cost. Some other control functions are also available on the bottom-right corner. With this human-machine interface, the weld quality inspection can be performed visually and remotely and the image data of the weld bead profile can be saved and output for further analysis.

## 4. Calibration of the Laser-Based Vision System

**Figure 4.**
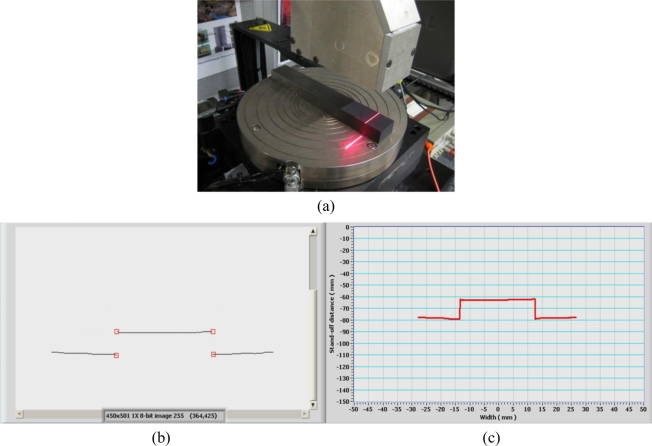
Calibration of vision system: **(a)** setup for the system calibration; **(b)** detected corner points of the reference coupon in the pixel space; **(c)** detected corner points of the reference coupon in the coordinate space.

Before performing measurement and weld quality inspection, the developed laser-based vision system needs to be calibrated first. There are several methods available for the camera calibration if taking many camera parameters into consideration. However, since this developed system not only consists of the camera, it also consists of other components such as the optical lens, optical filters. Besides these components, the arranged angles and distances between each component also greatly affect the final measurement accuracy of the system. So in this study, the developed vision sensor will be treated as a black box and the calibration is carried out directly based on the input actual geometrical features of a reference coupon and the output measured geometrical features of the reference coupon. A similar calibration method of the laser scanner is available in [18]. In order to do so, a reference coupon with a standard width of 25.4 mm and a thickness of 15.9 mm is used. As shown in Figure 4(a), the reference coupon is positioned perpendicularly to the projected laser stripe. In order to measure the stand-off distance, width and thickness of the coupon for calibration, the corner points of the reference coupon are extracted in the pixel space based on the first-derivative change of profile as shown in Figure 4(b). Then the detected corner points are transformed from the pixel space into the coordinate space by Equation (7) as shown in Figure 4(c). The stand-off distance as well as the width and thickness of the reference coupon then can be identified from the position information of the detected corner points in the coordinate space. In order to test and calibrate the performance of the developed vision system, the vision sensor moves vertically with the slide in the Z direction (as shown in Figure 2) from a stand-off distance of 50 mm to a stand-off distance of 120 mm at a speed of 10 mm/s. The frame rate of the camera is set to be 12 frames per second. Therefore, 84 continuous measurements of the stand-off distance, width, and thickness of the coupon are carried out.

**Figure 5.**
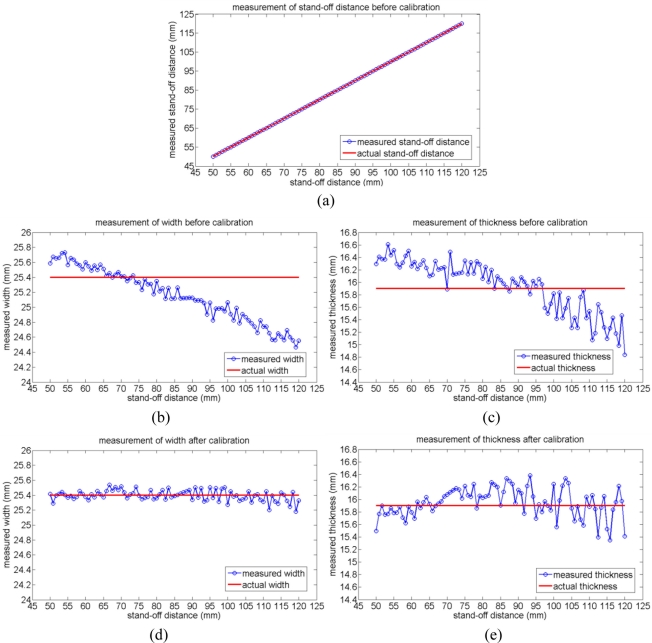
Calibration results of the laser-based vision system: **(a)** measurement of the stand-off distance before calibration; **(b)** measurement of the width before calibration; **(c)** measurement of the thickness before calibration; **(d)** measurement of the width after calibration; **(e)** measurement of the thickness after calibration.

As shown in Figure 5(a), the continuous 84 measurements of the stand-off distance between the vision sensor and the reference coupon before calibration agrees well with the actual stand-off distance when the vision sensor moves vertically. Therefore, there is no need to calibrate the system in terms of the stand-off distance measuremnt. However, as shown in Figure 5(b,c), the continuous measurements of the width and thickness of the reference coupon have some errors before calibration. The measuremnt errors change almost linearly with respect to the stand-off distance and the maximum error in terms of width measurement is up to 0.93 mm and the maximum error in terms of the thickness meansurement is up to 1.06 mm. In order to calibrate the system, two linear trendlines are calculated based on the method of minimum mean square error (MMSE). These two linear trendlines represent the approximately linear relationship between the measured width and thickness with respect to the accurately measured stand-off distance as shown in Equations (9,10), where *S_m_* is the measured stand-off distance, and *W_mt_* and *T_mt_* are the approximated width and thickness according to the calculated trendlines. As shown in Equations (11,12), the compensated values of the width and thickness, *W_c_* and *T_c_*, are calculated as the difference between the actual width or thickness of the reference coupon and the approximated corresponding values. These two compensated values of the width and thickness are then added to the actual measured width *W_m_* and thickness *T_m_* and then the calibrated measurements of the width *W* and thickness *T* are obtained as shown in Equations (13,14).

(9) $$W_{\mathrm{mt}}=-0.0166\times S_{m}+26.57$$

(10) $$T_{\mathrm{mt}}=-0.0175\times S_{m}+17.428$$

(11) $$W_{c}=25.4-W_{\mathrm{mt}}=0.0166\times S_{m}-1.17$$

(12) $$T_{c}=15.9-T_{\mathrm{mt}}=0.0175\times S_{m}-1.528$$

(13) $$W=W_{m}+W_{c}$$

(14) $$T=T_{m}+T_{c}$$

After calibrating the system, the continouous measurements of the width and thickness of the reference coupon are conducted again. As shown in Figure 5(d,e), the calibrated measurements of the width and thickness have maximum errors of 0.22 mm and 0.55 mm, respectively. It also can be observed that the measurement errors of the width and thickness increase when the stand-off distance increases. This is because the measurement resolution decreases when the stand-off distance between the vision sensor and the target weld increases. When the stand-off distance is 11.25 mm, the lateral and vertical measurement resolutions are 0.06 mm/pixel and 0.14 mm/pixel, respectively. And when the stand-off distance is 148.93 mm, the lateral and vertical measurement resolutions are 0.24 mm/pixel and 1 mm/pixle, respectively. Besides the factor of measurement resolution, the effects of refraction and distortion of the lens will also greatly affect the measurment accuracy when the stand-off distance is not proper. Therefore, in order to achieve a more accurate weld quality inspection, the stand-off distance between the vision sensor and the target weld should be in a proper range from 50 mm to 120 mm, in which range the measurement reuslts in lateral and vertical directions are acceptable for the purpose of weld quality inspection.

## 5. Results

**Figure 6.**
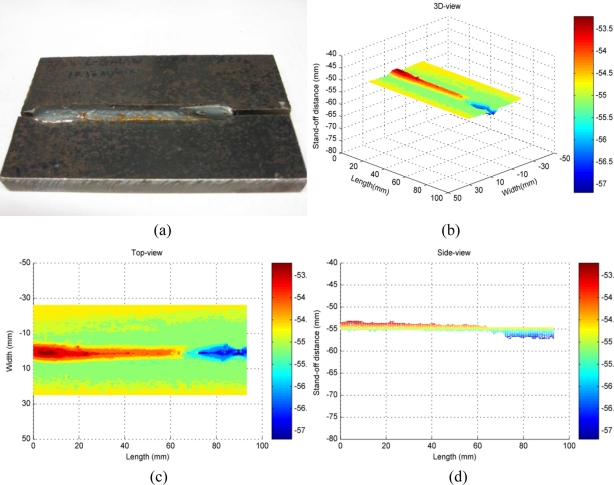
Results of weld quality inspection for laser-arc hybrid welding: **(a)** weld as the inspection target; **(b)** 3D-view of the inspected weld; **(c)** top-view of the inspected weld; **(d)** side-view of the inspected weld.

**Figure 7.**
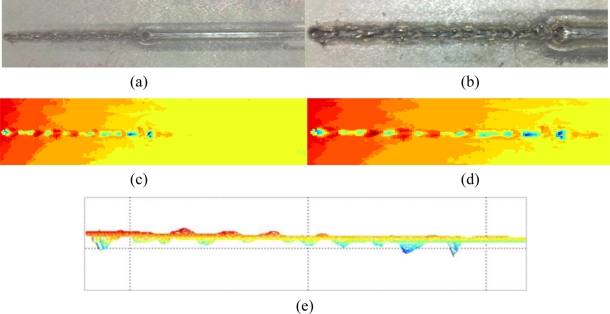
Results of weld quality inspection for laser welding: **(a)** weld as the inspection target; **(b)** detail of the weld; **(c)** top-view of the inspected weld; **(d)** detailed top-view of the inspected weld; **(e)** detailed side-view of the inspected weld.

After the calibration of the laser-based vision system is performed, the vision system is ready to be used for weld quality inspection. As shown in Figure 6(a), a weld was obtained by a hybrid fiber laser and gas metal arc welding of two thick metal plates. The profile of the weld bead is uniform at the beginning with minor reinforcement present. At the end of the weld, there is a weld defect present as incomplete filled groove. In order to obtain the 3D profile of this weld with the developed laser-based vision system, the worktable moves along the longitudinal direction of the weld so that the vision sensor can scan the full length of the weld bead at a constant speed. By scanning the top surface of the weld, the 3D profile of the weld is obtained as shown in Figure 6(b–d). The geometrical features of the weld are visualized by saving and outputting the image data into Matlab. The 3D view and top-view of the weld profile shown in Figure 6(b,c) clearly indicate the presence of the weld defect at the end of the weld bead. The side-view of the weld profile in Figure 6(d) shows the height change of the weld bead along the longitudinal direction. In order to further test the developed system for visual quality inspection of weld that has smaller details and weld defects, another weld coupon obtained by the laser welding of galvanized high-strength steel is used as the inspection target. The galvanized high-strength steel is widely used in the automotive industry due to its capability of high strength and corrosion-resistance that improve a car’s crash performance and service life. However, in a gap free lap joint configuration of galvanized metal sheets, the zinc vapor generated at the interface of the two metal sheets will cause the formation of spatters and blow-through holes. As shown in Figure 7(a) and its detailed view in Figure 7(b), many small spatters and blowholes are present at the weld. From Figure 7(c,d), it can be observed that these small spatters and blowholes are successfully detected by the developed vision system and different colors in the image represent different weld bead heights. As shown in Figure 7(e), the detail of the inspected weld from side-view shows the longitudinal profile.

**Figure 8.**
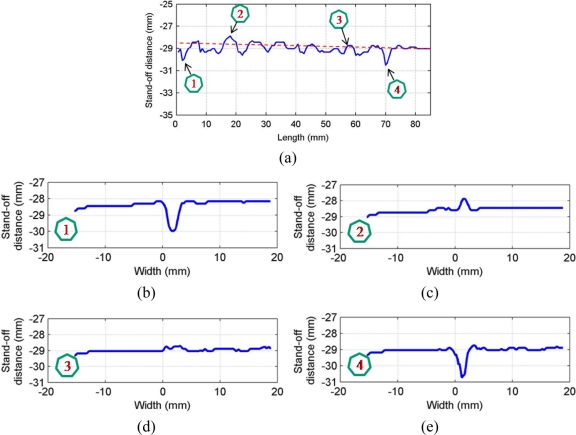
Geometrical evaluation of the inspected weld: **(a)** longitudinal profile of the weld; **(b)** traversal profile of the weld at point 1; **(c)** traversal profile of the weld at point 2; **(d)** traversal profile of the weld at point 3; **(e)** traversal profile of the weld at point 4.

By analyzing the geometrical features of the weld numerically, as shown in Figure 8(a), the longitudinal profile of the weld bead with respect to the top surface of the steel sheets (described by the red dashed line) is numerically available. By observing the longitudinal profile of the weld bead, the weld quality can be evaluated instantly. Also, as shown in Figure 8(b–e), the traversal profiles of the weld bead at four different points along the longitudinal direction are obtained. Based on this information along with the top-view of the weld bead, the different types of weld defects can be identified. For example, there is a blowhole or crater present in the weld bead with a diameter around 3 mm at the point 4 as shown in Figure 8(e). With these traversal profiles of the weld bead, an expert vision system based on pattern recognition can be further developed by comparing the traversal profiles of inspected weld with the template of the traversal profile of a sound weld. Chang *et al.* [19] employed a laser scanner to obtain the profile of a fillet weld and judged the acceptability of the fillet weld by comparing this obtained profile with the standard CAD section profile of a sound fillet weld. This method also can be used in this study to further automate the non-destructive weld quality inspection process and make it more intelligent.

## 6. Conclusion

In this paper, a non-destructive laser-based vision system is developed for the purpose of weld quality inspection. The vision sensor is designed based on the principle of laser triangulation. By designing the image processing modules, the images acquired from the vision sensor can be processed at a speed of more than 300 frames per second. By calibrating the laser-based vision system, the measurement resolution can be up to 0.06 mm/pixel laterally and 0.24 mm/pixel vertically. By scanning the weld bead with the developed vision system, its 3D profile can be obtained. From the obtained 3D profile of the weld bead, the presences, positions and sizes of the weld defects can be accurately identified. Compared to other 3D laser range sensors, there are several advantages of the developed system in this study that can be concluded as follows:

1. The system is developed based on LabVIEW, a platform with graphic-language based software packages and off-the-shelf hardware. These features enable the developed vision system to be more efficient both in terms of cost and time cycle for customized development and reconfiguration. Compared to other 3D range sensors available for off-line weld quality inspection, this new developed system is more suitable for industrial application because of its capabilities of on-line image processing and weld quality inspection in 2D and 3D space. This labVIEW based system can also be easily integrated into the automatic production line, which is of great importance for industrial welding applications.
2. Unlike some of the 3D range sensors that use complex image processing algorithms, the vision system developed in this study applies a relatively easy yet efficient and accurate algorithm to extract the laser stripe for inspection purpose. Besides that, the calibration method of the developed system is also easy to implement and the accuracy after calibration is adequate for the purpose of weld quality inspection.
3. The developed system uses the GigE camera as the image sensor. The unique features of the used GigE camera such as high image frame rate up to 196 fps and especially the long cable length up to 100 meters make it very suitable for welding applications in the industrial environment and easy integration with automatic production line.
4. The customized machine-user-interface offers different position information and geometry features of the detected weld surface and weld defects in real-time. The availability of the 2D and 3D profile of the scanned weld surface in real-time offers the potential for the system to be further developed with pattern recognition, which is able to identify different types of weld defects on-line. With other 3D range sensors, this cannot be achieved on-line.
5. One of the objectives of this study is to develop a cost-efficient system to perform the weld quality inspection. Compared to those expensive systems that are not suitable for real-time industrial applications as shown in Table 2, the cost of the laser-based vision system developed in this system study is much lower (as shown in Table 3). Besides its cost-efficiency, it also offers all the features that are necessary for real-time industrial application such as the on-line weld quality inspection.

## Figures and Tables

**Table 1.** Different types of weld defects.

crack 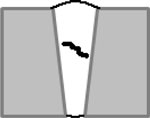	porosity 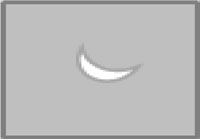	reinforcement 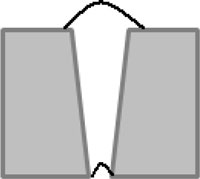 root concavity	undercut 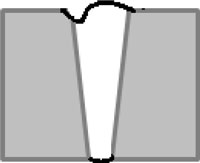
sharp corner 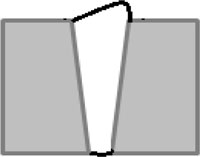	incomplete filled groove 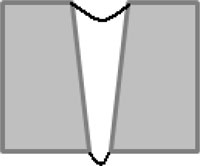 root dropout	misalignment 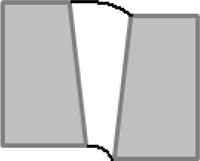	partial penetration 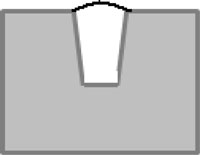

**Table 2.** Costs and features of different laser range sensors.

**system**	**cost (US dollar)**	**accuracy (mm) resolution (mm/pixel)**	**features**	**acquisition speed**
NextEngine laser scanner	2,995	0.0635–0.1693 mm/pixel	USB 2.0 cable (length limit 0.5 m)	2 minutes per scan 50,000 points/s
Surphaser HSX laser scanner	90,000 to 150,000	0.2 mm	360° × 270° field of view	800,000 points/s
DI3D 3D capture system	20,000 to 140,000	0.5 mm	Up to 21 mega pixels per image	
ViALUX z-Snapper 3D Camera	25,000 to 50,000	0.025 mm		Up to 40 fps
Konica Minolta Range 7 3D Digitizer	80,000	0.04 mm 0.08–0.28 mm/pixel	USB 2.0 cable	
Konica Minolta VIVID 3D Laser Camera	25,000 to 55,000	0.125 mm	can be used to scan people	
LDI SLP Laser Scanner	16,900 to 22,900	0.05625 mm		
FARO Laser ScanArm	30,000 to 40,000	0.035 mm		30 fps 19,200 points/s
FARO Laser Scanner Photon	90,000 to 120,000	3 mm	long range large volume scanner	
Geometry Systems, Inc. iScan	14,000 to 20,000	0.025 mm	link up multiple systems for simultaneous capture	
MicroScan 3D	15,000	0.2 mm	palm-sized scanner controller	
Kreon Zephyr Laser Scanner	60,000 to 75,000	0.025 mm		

**Table 3.** Costs and features of the system developed in this study.

**cost (US dollar)**	**accuracy (mm) resolution (mm/pixel)**	**features**	**acquisition speed**

Overall 2,500 camera 1,400 laser, optics 400 LabVIEW license 400 others 300	0.22 to 0.55 mm		
	0.06 to 0.24 mm/pixel (lateral)	GigE camera allows cable length to 100 m	up to 196 fps
	0.14 to 1 mm/pixel (vertical)	Real-time processing	up to 60,000,000 points/s
